# ‘Nurses as Gatekeepers’: Nurses’ Responses to Spiritual Needs of Patients with Primary Malignant Brain Tumors in Austria—Analysis of a Qualitative Vignette Study

**DOI:** 10.1007/s10943-025-02278-7

**Published:** 2025-02-21

**Authors:** Reinhard Grabenweger, Daniela Völz, Elisabeth Bumes, Megan Best, Piret Paal

**Affiliations:** 1 Institute of Nursing Science and Practice, Paracelsus Medical University, Strubergasse 21, Salzburg, 5020 Austria; 2 Department of Neurology and Wilhelm Sander - NeuroOncology Unit, Regensburg University Hospital, Regensburg, Germany; 3 Institute for Ethics and Society, University of Notre Dame Australia, Broadway, Australia; 4 Department of Ethnology, Institute of Cultural Studies, Tartu University, Tartu, Estonia; 5 Institute of Palliative Care, Paracelsus Medical University, Salzburg, Austria

**Keywords:** Spiritual care, Nursing, Neurosurgery, Brain cancer, Vignette study

## Abstract

This paper investigates the nurses’ gatekeeping role for spiritual care in multi-disciplinary teams as a way of responding to the spiritual needs of patients with brain tumors in Austria. Using a validated vignette in an online survey, qualitative data from 56 neurosurgical nurses (30.4% response rate) were analyzed via reflexive thematic analysis. One key theme, *Nurses as Gatekeepers – Referral and Working with other Health Care Professionals*, highlights nurses’ critical role in spiritual care. Effective referrals depend on nurses’ awareness of spiritual care specialists’ roles, emphasizing their importance in ensuring comprehensive, team-based care for neuro-oncological patients with complex spiritual needs.

**Supplementary Information:**

The online version contains supplementary material available at 10.1007/s10943-025-02278-7.

## Introduction

Seizures, cognitive deficits, drowsiness, and dysphagia are the four most prevalent symptoms of glioma (Ijzerman-Korevaar et al., 2018), the most common primary malignant brain tumor in adults, accounting for approximately 80% of all malignant brain tumors, with glioblastoma being the most common subtype (Schaff & Mellinghoff, 2023). Incidents of malignant brain tumors vary globally, with an average of 7 per 100,000 for all primary malignant brain tumors in the US, of which 80% are classified as gliomas (Schaff & Mellinghoff, 2023).

A wide range of symptoms negatively impacts the quality of life of glioma patients and changes across the disease trajectory from the diagnostic phase to the end-of-life phase (Ijzerman-Korevaar et al., 2018). All these complex symptoms are specific to gliomas because of their association with the central nervous system (Crooms et al., 2022). These neurological symptoms require specific management and care.

From the diagnostic phase onward, people with primary malignant brain tumors may lose their sense of identity, self-confidence, and their independence, which is experienced as a threat to their subjective well-being (Sutton et al., 2022). During the initial phase, defined as the period from symptom onset through biopsy or resection until diagnosis confirmation (Crooms et al., 2022), neurological and/or neurosurgical nurses take care of patients and have to deal with the complex symptom burden of these patients. The life-limiting glioma diagnosis and the complexity of symptoms that are associated with neurological deterioration necessitate early palliative care approaches, where specialist nurses are recommended for ongoing support and continual assessment of patients’ needs (Bozzao et al., 2023; Pace et al., 2017).

In the European Cancer Organization’s Essential Requirements for Quality Cancer Care (ERQCCs), specialist cancer nurses are required as core members of the multi-disciplinary team for adults with glioma. In European healthcare systems, patient-centered pathways and multi-disciplinarity are considered requirements for quality glioma care (Bozzao et al., 2023). Essential requirements for specialist nurses in neuro-oncology include the coordination of personalized treatment and support plans. Specially trained cancer nurses should establish trustworthy relationships with patients and their families (Bozzao & Weber, 2022). Additionally, nurses should incorporate the patient’s physical, psychological, social, cultural, and spiritual needs into their treatment and support plans to ensure needs-oriented and patient-centered care (Bozzao & Weber, 2022).

As long as the requirements for specialist cancer nurses are not fully implemented in neuro-oncological practice in European healthcare contexts, neurosurgical nurses could be considered as specialists who are embedded in a multi-disciplinary team. Concerning the patients’ and their families’ spiritual needs, neurosurgical nurses know about their spiritual needs and know how to meet these needs but do not always feel comfortable providing spiritual care (Nixon & Narayanasamy, 2010; Nixon et al., 2013). However, at the same time, relatives of patients in Sweden criticized nurses for failing to acknowledge patients’ spiritual needs. According to the interviewed nurses, obstacles to existential support were lack of time, not seeing it as their nursing duty, lack of knowledge and capacity, and the draining nature of the nursing job (Strang et al., 2001). This raises the question of neurosurgical nurses’ competencies and responsibilities concerning spiritual distress in patients and their caregivers.

## Background

In 1990, the World Health Organization (1990) published a report on cancer pain relief and palliative care, stating that spiritual needs should be acknowledged to relieve suffering. Suffering can be reduced by caring relationships respecting people’s diverse beliefs and offering supportive interventions which are “*non-sectarian, non-dogmatic and  in keeping with patients’ own views of the world”* (World Health Organization, 1990, p. 52). In accordance with these recommendations, spiritual care is defined as the following:

Spiritual care is that care which recognises and responds to the needs of the human spirit when faced with trauma, ill health or sadness and can include the need for meaning, for self worth, to express oneself, for faith support, perhaps for rites or prayer or sacrament, or simply for a sensitive listener. Spiritual care begins with encouraging human contact in compassionate relationship, and moves in whatever direction need requires. (NHS Education for Scotland, 2009, p. 6).

Spirituality was defined according to the European Association for Palliative Care (EAPC) (Nolan et al., 2011):

Spirituality is the dynamic dimension of human life that relates to the way persons (individual and community) experience, express and/or seek meaning, purpose and transcendence, and the way they connect to the moment, to self, to others, to nature, to the significant and/or the sacred. (p. 88)

While spiritual care is considered an integral aspect of palliative care, the context of our study focuses on neurosurgery, where nurses’ competencies in and resources for palliative care are limited. Nevertheless, all Austrian nurses – whether they work in a palliative care setting or not – are required by educational law to incorporate the spiritual dimension into their practice (Brandstötter et al., 2021). Whenever the term “*religious*” is used in this paper, it has to be noted that in an Austrian context with historically Roman-Catholic faith tradition, the term “*spirituality*” might be understood as the core of religion (Best et al., 2020). However, a conceptual analysis of the term “*spirituality*” from the perspective of Austrian nursing students revealed that spirituality is sometimes also clearly demarcated from religion (Paal et al., 2024). To be in line with the EAPC white paper the terms “*spiritual*”, “*religious*” and “*existential*” are understood as synonyms (Best et al., 2020).

Multi-disciplinary spiritual care provision should be incorporated into health care, and different models exist for integration (Balboni et al., 2014; Best et al., 2020). In palliative care, a multi-disciplinary model of spiritual care seems appropriate. Within this model, patients are seen holistically, and their spiritual needs are screened by all members of the healthcare team. However, due to the different levels of expertise in spiritual care, generalists like nurses, physicians, and therapists, need to be distinguished from spiritual care experts such as healthcare spiritual care workers (Best et al., 2020).

In this paper, we refer to the EAPC white paper on multi-disciplinary education for spiritual care in palliative care, where a healthcare spiritual care worker is defined as a “*staff member who has specialized in spiritual care within healthcare institutions, dedicated to SC [spiritual care] of patients, regardless of their religious background*” (Best et al., 2020, p. 3). According to this white paper the terms “*chaplain*”, “*pastoral care worker*”, “*board-certified chaplain*”, and *“(healthcare) spiritual care worker*” are used interchangeably.

The following recommendations have been made for all caregivers working in palliative care, while self-reflection is an important skill for all clinicians including nurses (Paal et al., 2024):

Staff needs to recognise the importance of spirituality in the life of the patient, and this requires a holistic approach, with the taking of a spiritual history and screening for spiritual need. In the event of spiritual distress, it is recommended that the patient be referred to a SC [spiritual care] specialist, that is, a trained health care SC worker, for personalised intervention. SC should be integrated into the patient and caregiver care plans, with initial assessment and ongoing interventions recorded clearly in the patient notes. (Best et al., 2020, p. 7)

Jones et al. (2020) have summarized the multi-disciplinary approach of spiritual care in a non-palliative, rehabilitation setting as: “*Spirituality is everybody*’*s business*". In contrast, however, Völz et al. (2024) have argued that when everybody is equally responsible for spiritual care, nobody may consider it their explicit duty and spiritual care might remain a neglected area of patient care. This highlights that there is still unclarity in the role perception of the multi-disciplinary healthcare team members in spiritual care provision, leading to our research problem.

Concerning nurses’ responses and their perceptions of the spiritual needs of patients, studies show that while some nurses are aware of the spiritual needs of their patients and have a general idea of how to respond, others feel insecure in their professional role and express uncertainty. Especially within secular societies some nurses do not consider spiritual care part of their job (Ghorbani et al., 2021; Nixon et al., 2013; Ramezani et al., 2014; Völz et al., 2024).

This leads us to the research questions of the NEUROSPIRIT-AT research project: (1) How do nurses respond to the spiritual needs of persons with primary malignant brain cancer in neurosurgical wards in Austria? (2) What are the nurses’ attitudes to the spiritual needs of persons with primary malignant brain cancer? We aimed to investigate neurosurgical nurses’ responses to possible spiritual needs of patients with primary malignant brain cancer and their attitudes when being confronted with these needs. The findings of this study should help improve holistic, patient-centered nursing care, which includes patients’ spirituality.

## Methodology

### Design

This was a cross-sectional, multicenter, qualitative vignette study using the online survey tool LimeSurvey. With this study design, we aimed to generate data with good *information power* (Malterud et al., 2016).

### Theoretical Framework

For this study, we chose a phenomenological framework based on Heidegger (1962). The phenomenological approach seemed appropriate for studying participants’ subjective experiences in their *lifeworlds* (Lebenswelt) while acknowledging our positionings as researchers in our *lifeworlds*.

### Study Setting and Recruitment

We collected data from registered nurses working in neurosurgical wards in Austria. To generate data from nurses working at different sites across Austria, managers of all neurosurgical departments were contacted and informed about the study. Seven of the 11 departments agreed to participate. Head nurses were asked to forward three emails to the nurses with the following aims: (1) to inform them about the study, (2) to ask for participation following a link to the online survey, and (3) to remind and thank them for participation.

### Inclusion and/or Exclusion Criteria

All registered nurses currently working in neurosurgical wards in Austria were eligible for inclusion in the study. The nurses did not receive any specific spiritual care training before their participation in the survey. Nursing assistants and other healthcare professionals were excluded, as we wanted to concentrate on registered nurses’ experiences. In addition, the chosen recruitment strategy made it impossible to reach undergraduate nurses who often did not have personal email accounts during their clinical internships. Nevertheless, we acknowledge the importance of every member of the multi-disciplinary team in spiritual care provision (Best et al., 2020; Jones et al., 2020).

### Data Generation

The data generated in this study were based on an online survey using one vignette as an eliciting tool appropriate for studying sensitive social phenomena, such as spirituality (Finch, 1987). The online survey mode seemed appropriate to study the phenomenon and to minimize undesired interviewer effects (Braun et al., 2020). However, we had no further influence on the sample size since we had to rely on the self-recruiting of the nurses who received an invitation to the online survey. We aimed for data generation from a sample which was homogenous concerning the Austrian healthcare context, but at the same time we sought for heterogeneity in age, gender, professional experience, extent of spirituality and religiosity. The survey was opened on the 7th of November and closed on the 4th of December 2022, after the friendly reminder emails were sent and the rate of response diminished rapidly. We used the online survey tool LimeSurvey for data collection.

Before participants were able to access the online survey, they had to read the study information with a short presentation of the spirituality definition we referred to in this paper (Best et al., 2020). Subsequently, the privacy policy was presented, and the participants were asked to give consent. A short sociodemographic questionnaire, including questions on self-reported spirituality and religiosity from the NERSH questionnaire (Hvidt et al., 2016), was presented. After that, one carefully constructed and validated vignette was used as a narrative stimulus, followed by open-ended questions (see Appendix 1: Vignette). The process of the vignette construction and validation has been previously described in a methods paper by Grabenweger et al. (2023).

The German equivalent study Neurospirit-DE used the same vignette to survey Bavarian neurological and neurosurgical nurses and physicians. The study′s findings demonstrated that the data generation method was reliable, as the analysis of the data brought similar insights to other surveys on spiritual care behaviors of healthcare professionals (Völz et al., 2024). By asking open-ended questions in addition to sociodemographic questions, we aimed to explore nurses’ responses and attitudes toward spiritual care (see Appendix 2: Original Survey in German). We had a special interest in nurses’ experiences with the spiritual needs of patients with brain tumors, which seemed coherent with our research questions and phenomenological research stance.

Prior to data analysis, the first author checked the data for integrity and verified whether participant anonymity was warranted. He used MAXQDA 2022 software for further qualitative data management. Quantitative survey data on participant characteristics were processed using IBM SPSS Statistics 27.

### Data Analysis

RG analyzed the data following *Reflexive Thematic Analysis (RTA)* by Braun and Clarke (2022), as this method allowed the analysis of the patterns of meaning in the data with respect to the research questions and the underlying epistemological position. In addition, the researcher’s reflexive subjectivity seemed very important in spiritual care research, where the reflexivity of the individual’s spirituality is a necessary prerequisite.

After grounding himself in the phenomenological philosophy of Martin Heidegger (1962), RG engaged with the data itinerating between the proposed phases for data analysis. Familiarizing himself with the data by reading and re-reading and taking notes, he first performed inductive and semantic coding. As a result of this process, the author recognized similarities to Heidegger’s philosophy and other spiritual care models, for example the *EPICC Spiritual Care Education Standard* (EPICC, n. d.) and the *ars moriendi model* by Leget (2017).

**Fig. 1:**
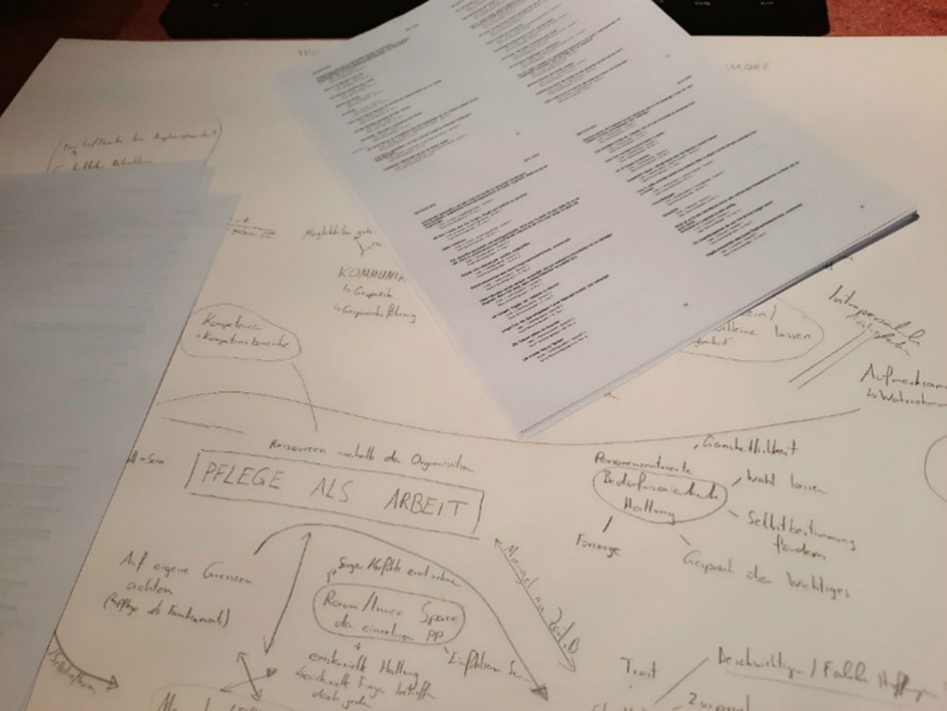
Initial Mapping of Themes

Consequently, he added deductive codes to the analysis. Finally, more implicit meanings were coded using latent codes. RG then generated initial themes by clustering codes to a shared patterned meaning (Fig. 1: Initial Mapping of Themes). By engaging more deeply with the data, candidate themes were developed, reviewed, and later refined, defined, and named. Finally, the research report was written to answer the research questions by telling RG’s story about the dataset. During the analysis process, the first author was in dialogue with his supervisor PP, an experienced spiritual care and qualitative researcher, to enhance the quality of the analysis.

The data analysis was done in German. Translation of the data extracts was initially done by using DeepL. After that the translated data extracts were checked by the research team including an English native speaker in order to get a nuanced translation (see Appendix 3: Original Data Extracts and their Translations).

### Ethical Considerations

The protocol was submitted to the appropriate ethics committee at the Johannes Kepler University Linz (1139/2022). The committee decided on June 8th, 2022, that no ethical approval was required because participation was voluntary, and the participants were not considered vulnerable. All participating hospitals gave permission to conduct the survey, and the survey participants gave informed consent. We adhered to the current data security policies of the European General Data Protection Regulation (GDPR). There were no ethical concerns at any point during the study.

### Rigor and Reflexivity

Concerning the rigor in reporting of this study, we followed Braun and Clarke’s *Reflexive Thematic Analysis Reporting Guidelines* (RTARG) (Braun & Clarke, 2024). The reflexivity of RG as an analyst was very important for performing the reflexive thematic analysis. Initially, RG started reflecting following Braun and Clarke’s strategy to begin reflexivity (Braun & Clarke, 2022). Thoughts on his social positioning, his role as a novice researcher, and his engagement with spiritual care were written down into a reflexive journal. The researcher used journaling to document his thoughts throughout the analytical phase. Researcher RG is a neurosurgical nurse in Austria and understands working conditions as well as spiritual care practices in Austria.

## Analysis

### Characteristics of Participants

Based on the feedback of those who circulated the invitation emails, we estimated that 184 nurses received the emails. A total of 70 nurses visited the online survey site and gave informed consent (38.0%), and 56 responses were included in the data analysis (30.4%). Incomplete datasets were also analyzed, explaining a certain number of missing data in Table 1. The characteristics of the online survey participants are presented in Table 1. In our survey, which focused on qualitative data generation, the nurses were asked for their professional experience in general. This does not allow drawing any conclusions on the possible correlations between nurses’ specific experiences with glioma patients and their reported behaviors.

**Table 1:** Participant Characteristics

Nurses (N = 56)	
*Gender*	n (%)
Female	34 (85.0)
Male	6 (15.0)
*Age (years)*	
21–30	9 (23.7)
31–40	8 (21.1)
41–50	9 (23.7)
51–60	12 (31.6)
*General professional experience (years)*	
0–10	11 (29.7)
11–20	6 (16.2)
21–30	11 (29.7)
31–40	9 (24.3)
*Self-reported spirituality*	
Very spiritual	4 (10.3)
Moderately spiritual	21 (53.8)
Slightly spiritual	11 (28.2)
Not spiritual at all	3 (7.7)
*Self-reported religiosity*	
Very religious	5 (13.2)
Moderately religious	14 (36.8)
Slightly religious	16 (42.1)
Not religious at all	3 (7.9)

### Generated Themes

**Fig. 2:**
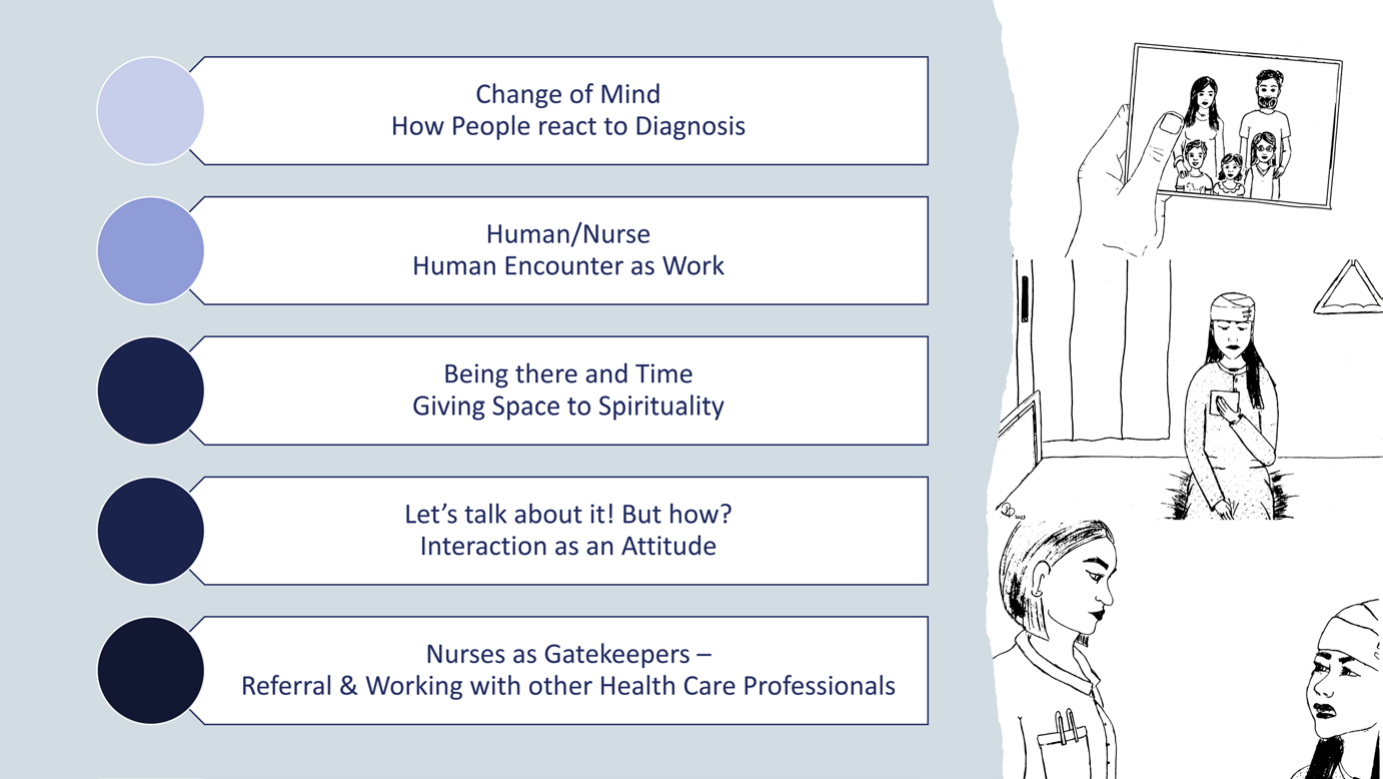
Generated Themes

The first author generated five themes in his data analysis. According to Braun and Clarke (2022), a theme was understood as “*a pattern of shared meaning organised around a central concept*” (p. 77). As this paper concentrates on the theme *Nurses as Gatekeepers – Referral and Working with other Health Care Professionals,* we first present a short overview of the themes to get an introduction to the analytic story (Fig. 2: Generated Themes). After that we report on the theme *Nurses as Gatekeepers – Referral and Working with other Health Care Professionals* in detail, as we believe that an in-depth exploration of this theme is essential to emphasize the critical role nurses play in the provision of spiritual care. This paper is dedicated to a thoroughly analysis of this theme, allowing ample space for analytical depth and a comprehensive discussion.

Following five intertwined themes were generated: (1) Change of Mind – How People react to Diagnosis; (2) Human/Nurse – Human Encounter as Work; (3) Being there and Time – Giving Space to Spirituality; (4) Let’s talk about it! But how? – Interaction as an Attitude; and (5) Nurses as Gatekeepers – Referral and Working with other Health Care Professionals (Table 2: Themes).

**Table 2:** Themes

Themes	Theme description	Data extracts
Theme 1: Change of Mind – How people react to diagnosis	Patients diagnosed with malignant brain tumors react differently to the changed life situation after diagnosis. Reactions could be emotionally intense, which illustrates the relevance of spiritual care provision on neurosurgical wards.	*Yes, there are always situations in which patients, relatives and caregivers ask themselves "Why does it happen to me? /Does it happen to her/him?". In inpatients’ everyday life, there was not explicitly a young mother with three children. But a young father or patients who were diagnosed shortly after retirement and actually wanted to "enjoy" life once again. (65, pos. 1)* *I had many defusing conversations with the patient and helped her to understand the information from the doctor and was constantly available to answer questions [...].* *The feeling of not being alone and not getting lost in the whirl of feelings and information and also allowing time to process the feelings and impressions. (5, pos. 2–3)*
Theme 2: Human/Nurse – Human Encounter as Work	Nurses are persons with their own human needs who try to meet the patients’ needs in a professional and holistic way. It may create tensions because of working conditions and nurses’ vulnerability.	*Yes, I have been several times, when I started my job in 2007 I was always very sensitive in these situations myself and had to be careful not to cry with the patients, but in the meantime I have learned to isolate myself and see work as work. Nevertheless, I still find it difficult, especially when the patients are the same age as me or younger and previously had a happy and satisfying life. (62, pos. 1)* *After the patient refused my suggested help and I still had to go to the next room for time reasons, I assured her that she could contact me at any time if she needed anything. (24, pos. 1–2)*
Theme 3: Being there and Time – Giving Space to Spirituality	Presence and availability, time and “inner space” or capacity for spiritual care are important requirements for adequate care of neurosurgical patients with spiritual needs.	*Such events occur relatively often in my everyday working life.* *However, in my experience, it also depends very much on how much space you as a person/nurse allow for such situations.* *In other words, how much sensitivity, beyond visible conditions, and empathy I bring with me and also signal non-verbally a willingness to talk. (47, pos. 1–3)* *Signaling willingness to talk, take time, give time, let people talk (27, pos. 3)*
Theme 4: Let’s talk about it! But how? – Interaction as an Attitude	Nurses are interested in communicating with patients at eye level. Certain communication skills are important to recognize patients’ needs – especially when they suffer from speech difficulties.	*I try to meet the patient at eye level and listen carefully to their concerns, offer psychological support if necessary and organize it as soon as possible. It is usually very valuable for the affected person to have someone to talk to who takes them seriously.* *Honesty is very important in this conversation.* *A contact person who takes the time to listen to their concerns (10, pos. 1–3)* *- if she allows it - sit down with her, talk to her - or sometimes say nothing (26, pos. 7)*
Theme 5: Nurses as Gatekeepers – Referral and Working with other Health Care Professionals	Nurses who recognize spiritual needs refer patients to other members of the multi-disciplinary team. Nurses play an important role as the interface between patients, various other healthcare professions and the family. Working together in the multi-disciplinary team can be experienced as a relief for nurses.	*I think it‘s important that the nurse doesn‘t feel overwhelmed by the conversations and can also withdraw.* *Other professional groups such as psychologists and psychiatrists are always involved at the patient‘s request and reduce the burden for the nursing staff. (55, pos. 11–13)* *referred to doctors to get psychol. support (39, Pos. 1)*

#### Change of Mind – How People React to Diagnosis

In the first theme *Change of Mind – How People react to Diagnosis*, the reactive patterns of people with suspected or diagnosed malignant brain tumors in neurosurgical wards were identified. The extent to which spiritual needs can be expressed in an inpatient setting is apparent in the reports of the surveyed nurses. Different types of responses were identified in this study if nurses perceived the spiritual needs of the patients. These responses were analyzed in the following themes:

#### Human/Nurse – Human Encounter as Work

It was discernible that nurses wanted to meet spiritual needs showing compassion, which can, however, be experienced as stressful by individual nurses. Sometimes, nurses meet the needs of patients in the role of task-oriented caregivers. Nurses then limit themselves to known nursing interventions or clearly define their area of competence for themselves, which means that the patients’ needs cannot always be adequately addressed.

#### Being There and Time – Giving Space to Spirituality

The title of this theme alone strongly highlights the connections with Heidegger’s phenomenological thoughts which built the theoretical framework of this analysis. Space and time also appear to be essential prerequisites for perceiving these personal and individually different needs. Giving space can be understood here as the capacity of nurses to be open to the individual needs of the people being cared for, and to consciously perceive these needs. However, time is required to adequately meet these needs, both to allow the patient with possible communication problems to articulate their problems as well as to address them.

Within the neurosurgical setting, this means that the attitudes of nurses are strongly influenced by their working environment, which can be understood as their *lifeworld* in a phenomenological understanding. Limited time and resources make it difficult to include spiritual care in nursing practice on a busy ward. Nevertheless, the presence and round-the-clock availability of nurses can be seen as an advantage in spiritual care provision.

#### Let’s Talk About It! But how? – Interaction as an Attitude

In neurosurgical wards, nurses attempt to interact with spiritually distressed people through conversations or non-verbal communication to meet their spiritual needs. Nurses follow known principles of conversation such as active listening. The focus here is often on the diagnosis of needs within the family systems and on possible referrals within the multi-disciplinary team in the inpatient setting.

#### Nurses as Gatekeepers – Referral and Working with other Health Care Professionals

The organization of therapies or medical interventions by making referrals was the central organizing concept of this theme. Nurses play a mediating role within the multi-disciplinary team to facilitate holistic care. Nurses can therefore assume the role of gatekeepers for spiritual care provision.

### Focused Theme: Nurses as Gatekeepers – Referral and Working with other Health Care Professionals

This theme combines two sub-themes under the overarching topic of mediation: (1) cooperation with other professional groups and (2) mediation within family systems. Nurses play an important role as they interface with patients, various healthcare professionals, and patients’ families. However, care must be taken to ensure that spiritual needs or crises are not pathologized by rapid referrals to psychiatrists and psychologists. An important prerequisite for the mediation of other professional groups or in the families is the competence to identify the respective needs as spiritual needs and to make an adequate assessment. Some nurses think and act holistically by considering the social system of their patients when providing person-centered care. Working together with other professional groups can be experienced as a relief for nurses.

**Fig. 3:**
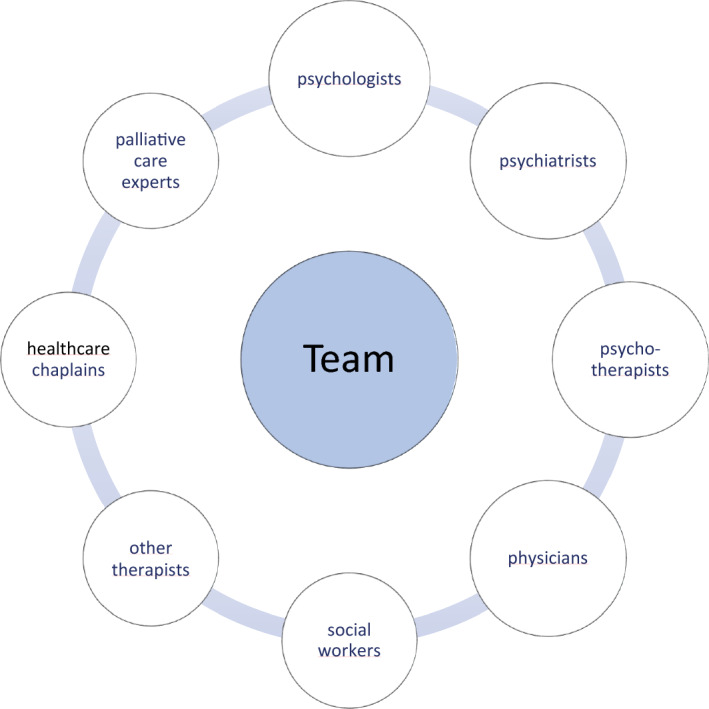
Referrals to other multi-disciplinary team members

Nurses are aware of their position in multi-disciplinary teams and value the benefits of teamwork. In the survey, nurses mentioned referring their patients to members of the following healthcare professions (Fig. 3: Referrals to other multi-disciplinary team members): psychologists, psychiatrists, psychotherapists, physicians, social workers, pastoral care workers/chaplains, therapists, people from the psychosomatic consultation-liaison service (C/L service), and nursing colleagues with special knowledge of palliative care or psycho-oncological support. This diversity of disciplines alone reflects the large multi-disciplinary team in the inpatient setting, which aims to provide holistic care for patients.

When it comes to nurses’ reported referral behaviors, two main ways for referring patients to these professional groups were identified: (1) either simply calling them without consulting the patient, or (2) suggesting or offering to organize contact with these healthcare providers and letting the patient decide.

The first referral behavior shows that the patient is deprived of choice. For example, quickly arranging contact with psychiatrists and psychologists can lead to bypassing patients and to a rejection of the offer by the overwhelmed patient. The following quotes indicate that the patient is not always consulted before referral making.

Referred to doctors to get psychol[ogical] support (39, pos. 1)

After the interview, I would contact the doctor to give her something reassuring if necessary. (64, pos. 3)

+ Consult psychological services or specially trained persons incl. social work (2, item 5)

By these rapid referrals without consultation with the patient spiritual distress may risk being pathologized, even though, in certain life situations, it is a natural response to feel temporary distress and ask existential questions - especially during a serious health crisis.

However, spiritual distress can also go beyond the norm, being pathological and requiring urgent treatment, so that help of a physician (e.g. psychiatrist) may be necessary. In these cases, nurses consider it necessary to refer patients to psychiatrists, as the following examples show.

She refused medication, psychological and psychiatric consultation took place the next day. (46, pos. 3)

Later, the psychologist and/or psychiatrist can be consulted. (55, Pos. 10)

In this context, nurses seemed to assume pathological changes and felt that medical treatment was necessary to release the patients from spiritual suffering. Nevertheless, healthcare professionals must critically evaluate the type of treatment needed and consider what should be offered to the patients.

Concerning the second referral behavior, other nurses seemed to be very reflexive and explicitly reported the offers they made to patients. They offered psychological support, psychotherapy, etc., and recommended contacts but gave patients the choice in advance as to whether they wanted these people to know about their personal crises.

Offer psychological support if necessary and organize this promptly (10, item 1)

Clinic social work and psychology are offered and called in if necessary (36, Pos. 2)

Encourage patients to make use of professional counseling (CL service) (61, Pos. 1)

Furthermore, some nurses reported contact with and referrals to chaplains. It appears crucial for the surveyed nurses to have a clear understanding of the individual’s religiosity. These data extracts of nurses mentioning chaplains show that nurses only think about pastoral care when they identify patients as religious, as the following quotes demonstrate.

If the patient is religious to contact pastoral care. (61, Pos. 3)

Offer of psychology and/or pastoral care (if the patient is religious), (27, Pos. 1)

Responses from nurses in the online survey did not indicate any referrals of patients with spiritual needs — beyond those explicitly recognized as religious — to spiritual care experts, such as healthcare spiritual care providers.

The data analysis showed that nurses need information about the person to be cared for before they can organize contact with other members of the multi-disciplinary team, but they also need to know how to diagnose a spiritual problem if they are to refer appropriately. Being informed is a prerequisite to being able to act in a person-centered and needs-oriented manner. Detailed medical and/or spiritual history taking and nurses’ ability to identify the patients’ needs appear to be important factors to ensure the best possible care.

Another point that must be mentioned in connection with working in a multi-disciplinary team is the relief provided to the nursing staff when other professionals are available. According to the nurses’ answers, professionals with special knowledge of psychosocial or spiritual support can respond better to certain patient needs than nurses themselves and thus avoid overburdening nursing staff. The following quotes explicitly illustrate this aspect:

Our job is[,] to listen and to involve the psychologist working clinically on the ward if we are overwhelmed. (55, item 6)

I think it’s important that the [nurse] doesn’t feel overwhelmed by the conversations and can also withdraw.

Other professional groups such as psychologists and psychiatrists are always involved at the patient’s request and also relieve the nursing staff. (55, pos. 11–13)

Overall, the importance of multi-disciplinary collaboration in the care of people with spiritual needs is emphasized by the statements of the nurses. This division of labor can lead to a reduction in the workload of the nursing staff, thus allowing better care for patients.

## Discussion

After generating the five themes that tell our story about spiritual care for people with primary malignant brain tumors, the focused theme *Nurses as Gatekeepers – Referral and Working with other Health Care Professionals* is discussed in reference to the existing literature. Although the focused theme and the selected data extracts must not be considered in isolation from the other generated themes, two questions arise: (1.) What is the nurse’s role in the multi-disciplinary team when it comes to spiritual needs of the patient? (2.) Is making referrals sufficient to address the spiritual needs of patients in nursing care?

### Nurses’ Gatekeeping Role

The theme *Nurses as Gatekeepers* clearly shows that nurses play an important role in multi-disciplinary teams caring for patients with primary malignant brain cancer. When it comes to the integration of the spiritual dimension in care, nurses must understand their own and other professionals’ roles in providing spiritual care, which is explicitly defined in Competence 3 of the Enhancing Nurses’ and Midwives’ Competence in Providing Spiritual Care (EPICC) project (McSherry et al., 2021). This is an important prerequisite for adequate referrals. In terms of the number of professionals named by the nurses in the survey, it can be concluded that nurses are aware of a range of healthcare professionals who can address patients’ spiritual concerns (Fig. 3: Referrals to other multi-disciplinary team members).

However, it must be questioned whether referrals to members of the psychosocial team or even to psychiatrists are the most appropriate referrals in cases of patients’ spiritual distress. In this context, nurses might make better referrals if they knew about the role of pastoral care practitioners in hospital wards and felt the chaplain’s presence in the multi-disciplinary team (Best et al., 2022; Van Leeuwen et al., 2006).

Besides knowing about the multiple professional roles, nurses need to know about the concepts of spirituality and religion. An Austrian nursing student cohort indicated that spirituality is a fragile concept, which interlinks with the potential tenuousness of nurses’ spiritual well-being, with terms such as hopelessness, meaninglessness, and lack of stability. Furthermore, demarcation from religion was pointed out (Paal et al., 2024). Best et al. (2020) have already highlighted the issue that spirituality is often incorrectly assumed to be exclusively religious. As a consequence of this misunderstanding, patients who identify themselves as non-religious may refuse consultations with chaplains.

During analysis the authors asked themselves following questions: Do nurses recognize spiritual distress as such if they classify the patient as non-religious? And, in contrast, how do nurses respond to distress that is related to religion? There is a risk that spiritual distress in patients may be misinterpreted as e.g. psychosocial problems and therefore treated inappropriately. Lacking knowledge may lead to a confusion of spirituality with religion. In this case, then, nurses will use their powerful gatekeeping role to block access to chaplains or other spiritual care specialists.

### Making Referrals - Is this Enough?

Some nurses who are members of a multi-disciplinary team experience relief when they are overwhelmed by patients’ spiritual needs and/or their workload and can refer to another discipline. In professional and ethically correct spiritual care provision, the recognition of personal limitations is essential (Giske et al., 2021). Nurses’ self-awareness of personal limitations is important for early and adequate referrals to specialists, such as healthcare chaplains and spiritual advisors.

At this point, it must be noted that when making referrals to spiritual care specialists, the patient’s privacy and confidentiality in the nurse-patient relationship require respect, as a person’s spirituality can be a very personal and intimate aspect. Continuous self-reflecting behaviors enhance nurses’ awareness of their spiritual care capacities, as also defined in Competence 1 of the EPICC network (Van Leeuwen et al., 2021). If the nurse feels overwhelmed by the spiritual distress of the patient, self-reflection and exchange with other people such as colleagues, members of the healthcare chaplaincy team, psychosocial team, as well as proficiency in spiritual care were identified as strategies to reduce compassion fatigue (Baqeas et al., 2021; Pierdziwol, 2022).

When it comes to collaboration with and referrals to other members of the multi-disciplinary team, nurses have a privileged position in patient care (Caldeira et al., 2019), characterized by presence and continuity, which can be seen as advantageous in providing spiritual care. Clarke (2013) demonstrated that spiritual care can also be seen as an integral part of everyday nursing practice. The integration of non-verbal spiritual care interventions into common nursing practice like helping somebody to eat or bathe can be seen as a possible way of spiritual care provision to patients with communication difficulties such as aphasia (Weegen et al., 2020).

In reference to nurses’ referral behavior Baldacchino (2006, p. 887) clearly stated that “*[…] the nurse should take an active role in meeting patients’ spiritual needs and not simply referring them to a chaplain.”* Therefore, it is not enough to merely refer patients to spiritual care specialists who deal with finding meaning and purpose in life and/or religious needs of patients, such as chaplains, psychologists, or social workers (Baldacchino, 2006).

If rapid referral of patients occurs, the relationship between patient and nurse could be negatively impacted, which may result in loss of the patient’s trust in the nurse (Cone & Giske, 2022). Patients may even feel rejected by the nurse when they are referred to others too quickly. In conclusion, it seems beneficial to the nurse-patient relationship if nurses are aware of their capacities and show compassion and presence. In instances where they perceive that they have reached their limits, they should be able to refer sensitively and ethically to other professionals.

### Strengths and Limitations of the Work

This study has some methodological limitations that influenced our analysis. First, we presented a typified situation in the survey vignette, which meant that we did not choose an overly dramatic situation. In addition, the vignette included no hints of the patient’s religiosity, such as a rosary, a cross, a woman’s veil, or other religious symbols. The existential question “*Why me?”* represents a universal human question, but could have influenced the nurses to be less likely to mention chaplains as experts for spiritual needs, because of nurses associating chaplains with religion.

When it comes to transferability, it must be noted that the presented themes are generated with data from Austrian registered nurses and are therefore contextualized knowledge. By presenting the participant characteristics in Table 1, transferability to other contexts is possible to some extent.

Regarding the iterative process of data analysis, it must be highlighted that the first author was a novice qualitative researcher and did his best to integrate as much reflexivity as possible in his research process. The importance of the researcher’s reflexivity is be seen as a strength in spiritual care research because reflexivity itself is also an important competence in spiritual care. Moreover, the first author is a neurosurgical nurse and thus has first-hand experience with the working conditions and care principals in this specialized field, which aligned well with the theoretical framework of this paper which was based on Heidegger’s Phenomenology. Thus, he could deepen his analysis through his reflexive research practice.

### Recommendations for Further Research

Further research is necessary to better understand the importance of spirituality in patients with severe neurooncological diseases and their families. Rapid neurological deterioration and cognitive changes might greatly influence the experience of spirituality in this patient group, as shown in a neurobiological study (Ferguson et al., 2022). This means that spiritual needs might change very quickly, for example before and after surgery. Awareness of the dynamic nature of spirituality is important for the assessment of spiritual needs, which once more underlines that spiritual assessment is a continuous process (McSherry et al., 2021). More evidence on appropriate assessment in this patient group with complex neurological symptoms, such as disorientation and speech disorders, is needed (Völz et al., 2024).

### Implications for Policy and Practice

The study’s analysis showed that nurses in neurosurgical wards, comparable to other acute care wards, play an important role in the integration of patients’ spiritual needs in care pathways. The value of specialist cancer nurses in multi-disciplinary teams must be strengthened as, for example, the European Cancer Organization stated in its paper on essential requirements for quality cancer care of patients with glioma (Bozzao & Weber, 2022).

To be able to address spiritual needs, nurses would benefit from spiritual care training, which invites them to reflect on their own spirituality and develop awareness of the spiritual dimension of health and illness (Jones et al., 2021; Paal et al., 2024). International and national policies should guarantee adequate spiritual care training by including it in nursing curricula, which is very heterogenous in Europe (Brandstötter et al., 2021; McSherry et al., 2020).

Besides the importance of spiritual care training, it must also be acknowledged that spiritual care giving capacities might develop within the professional career of nurses (Giske & Cone, 2015). The acute neurosurgical setting is demanding. Therefore, self-care competencies and coping mechanisms are important for nurses. A study on the development of coping skills in palliative care nurses showed that the ability to care for patients holistically develops with a professional career, which might also be of great relevance for neurosurgical nurses caring for patients with severe illnesses (Arantzamendi et al., 2024).

Based on our analysis, the following implications can be presented for nursing practice:

- Nurses need to know their role and the roles of spiritual care specialists in providing spiritual care at their workplaces.
- Nurses need to know the spiritual care specialists in their organizations; in the best case, they know them personally. It must be clear how to contact the spiritual care specialist and contact hours should be defined.
- Nurses need space and time for self-reflection and the development of self-care competencies and coping mechanisms. Nurses must know their personal limits and be able to refer sensitively and ethically to other professionals.
- Nurses should be encouraged to show presence and compassion towards patients, even in demanding settings, such as acute care.
- Nurses must remember their privileged position in patient care, which should be characterized by trustful patient-nurse-relationships.

## Conclusions

Our analysis showed that nurses play an essential role in providing spiritual care in acute neurosurgical settings. Nurses are seen as gatekeepers to spiritual care experts, such as healthcare chaplains, spiritual advisors, and other members of the multi-disciplinary team. It is important for nurses to know about their privileged role in relationship-building, which is characterized by compassion and presence.

In view of their powerful position in spiritual care, nurses should know how to assess spiritual needs and make adequate referrals with patients’ permissions in order to provide the best possible care. Nurses have to know about the different roles and competencies of other members of the multi-disciplinary team, which can vary from hospital to hospital or from country to country. Multi-disciplinary spiritual caregiving depends on nurses’ capacities and competencies, clearly highlighting the importance of spiritual care training for nursing professionals.

## Supplementary Information

Below is the link to the electronic supplementary material.

Supplementary file1 (DOCX 19 KB)

Supplementary file2 (DOCX 988 KB)

Supplementary file3 (DOCX 20 KB)
