# Supplementary material for: ‘Nurses as Gatekeepers’: Nurses’ Responses to Spiritual Needs of Patients with Primary Malignant Brain Tumors in Austria—Analysis of a Qualitative Vignette Study
Source: J Relig Health. 2025 Feb 21;64(2):732–53. doi: 10.1007/s10943-025-02278-7 (PMC11950041; doi:10.1007/s10943-025-02278-7)
Supplement: Supplementary file 1 — Supplementary file1 (DOCX 19 KB) [file 10943_2025_2278_MOESM1_ESM.docx]

Appendix 1: Vignette

You are on night duty and come to see Ms. S. on your ward in the evening. You find her hunched over in bed. You ask Ms. S. about her condition.

Ms. S. is 38 years old, married and the mother of three children (4, 6 and 9 years old). A few days ago she was admitted to the hospital with a mild, right-sided weakness and a speech disorder. She has noticeable difficulties communicating verbally. She was told by the doctor that she had a large, fast-growing, and very likely malignant brain tumor. A diagnostic biopsy was performed. She is waiting for the histological result.

You approach Ms. S. and see that Ms. S. is holding a photo of her children in her left hand. Apparently she got this photo today. She looks at it, shakes her head, tries to say something, and only produces snippets of words at first. Ms. S. seems hopeless and tearful. Then she utters clearly: "Why me?"

**Open-ended Questions:**

1) Please tell us from your personal clinical practice. Have you ever experienced a similar situation? How did you react?

2) What do you think is most important to Ms. S. in this situation?

3) How could you help Ms. S. in this situation?
