# Supplementary figures and images for: ‘Nurses as Gatekeepers’: Nurses’ Responses to Spiritual Needs of Patients with Primary Malignant Brain Tumors in Austria—Analysis of a Qualitative Vignette Study
Source: J Relig Health. 2025 Feb 21;64(2):732–53. doi: 10.1007/s10943-025-02278-7 (PMC11950041; doi:10.1007/s10943-025-02278-7)

Appendix 2: Original Survey in German


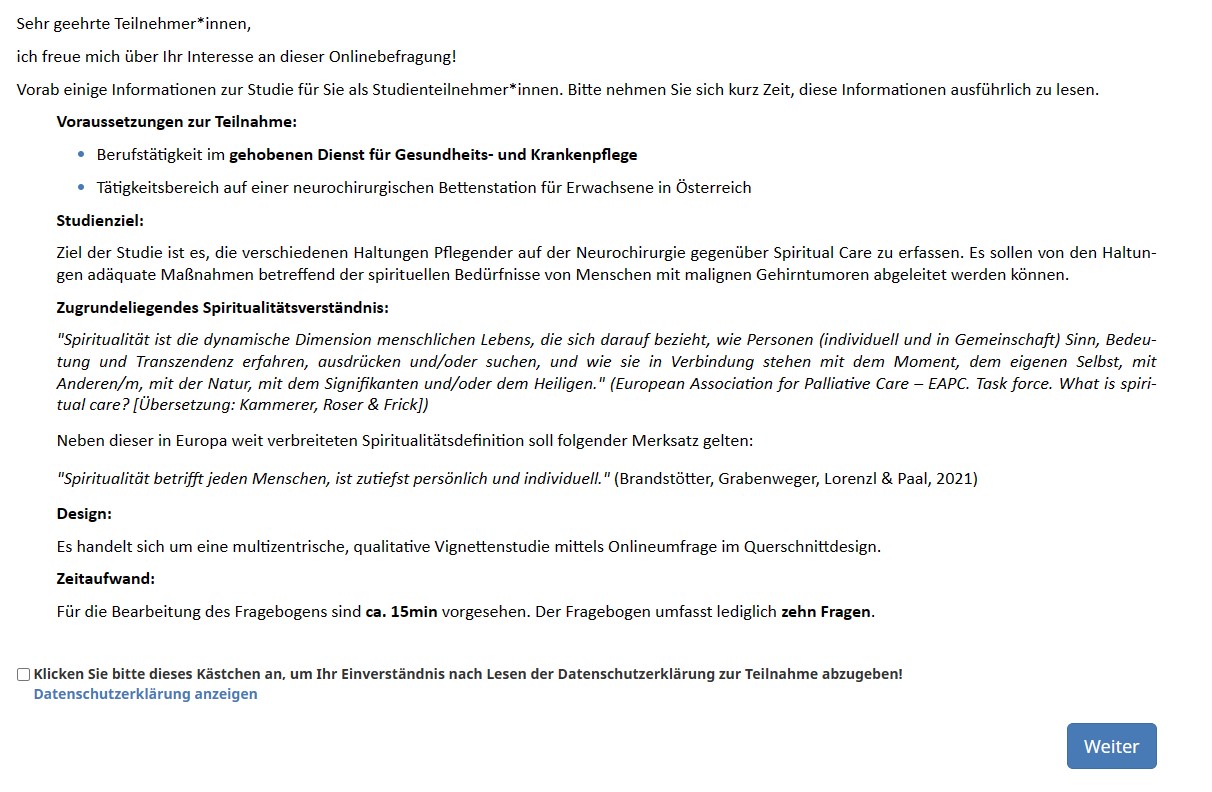


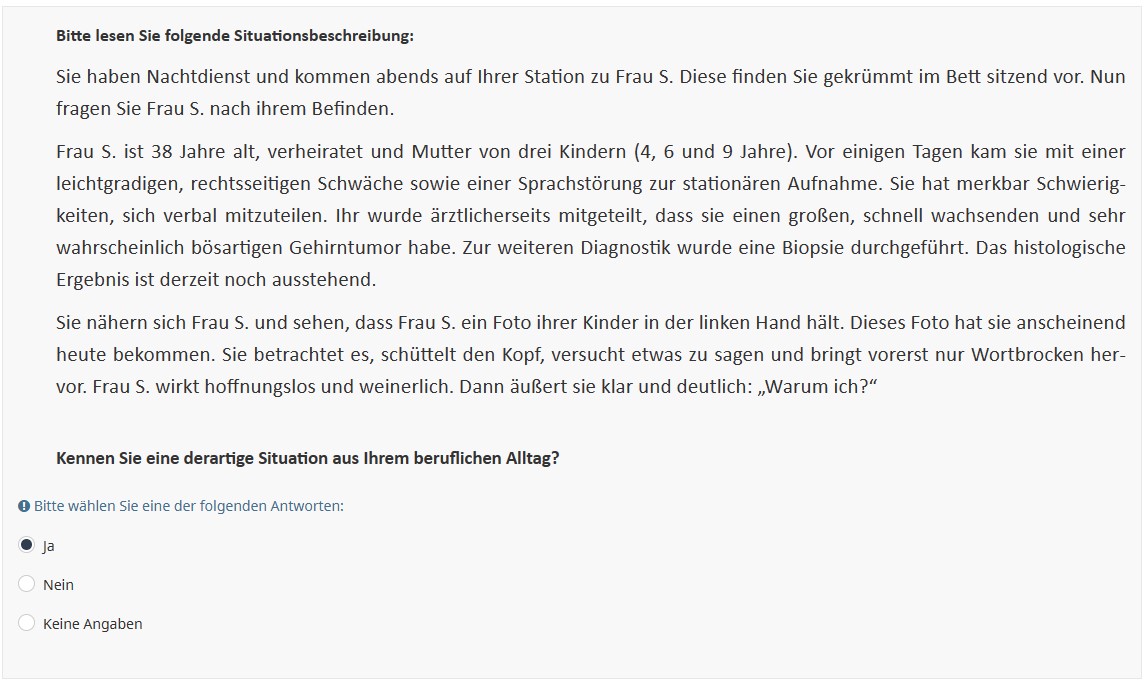

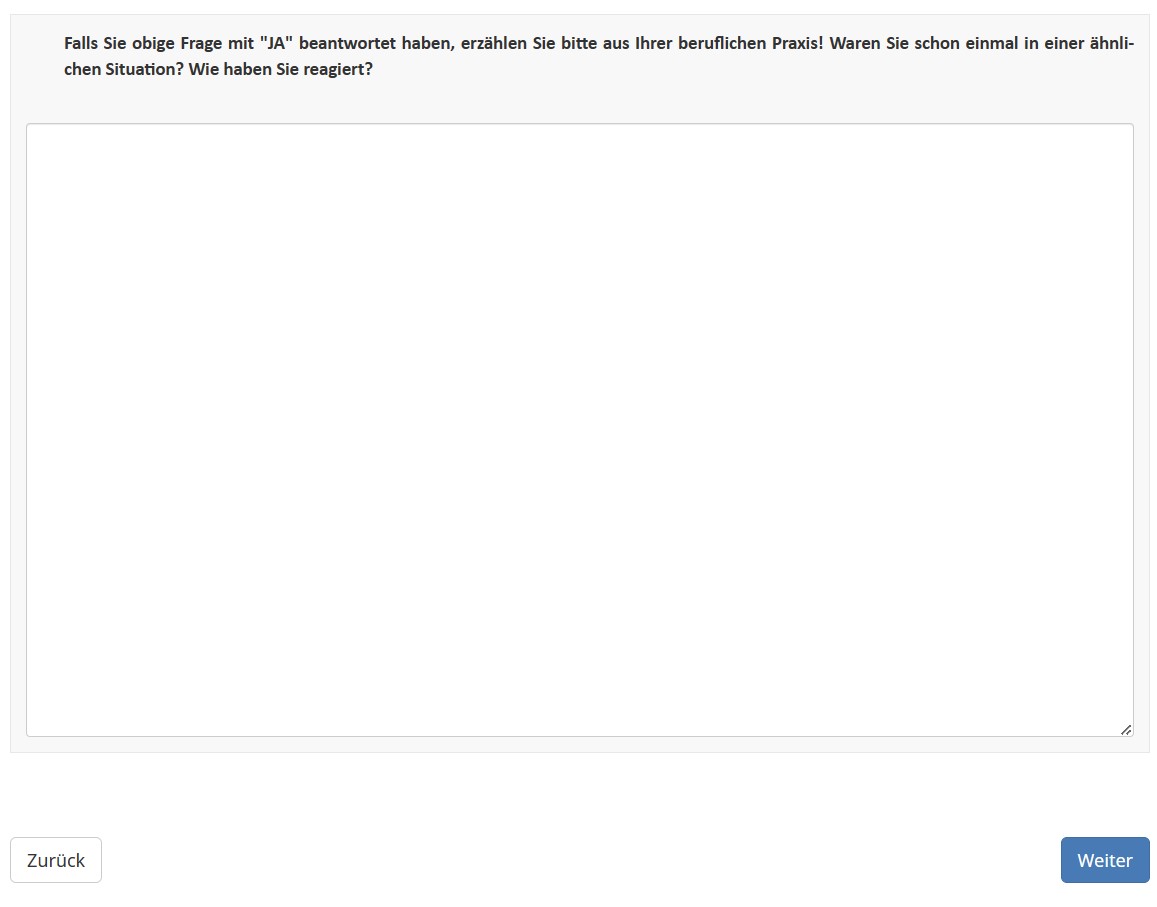


**
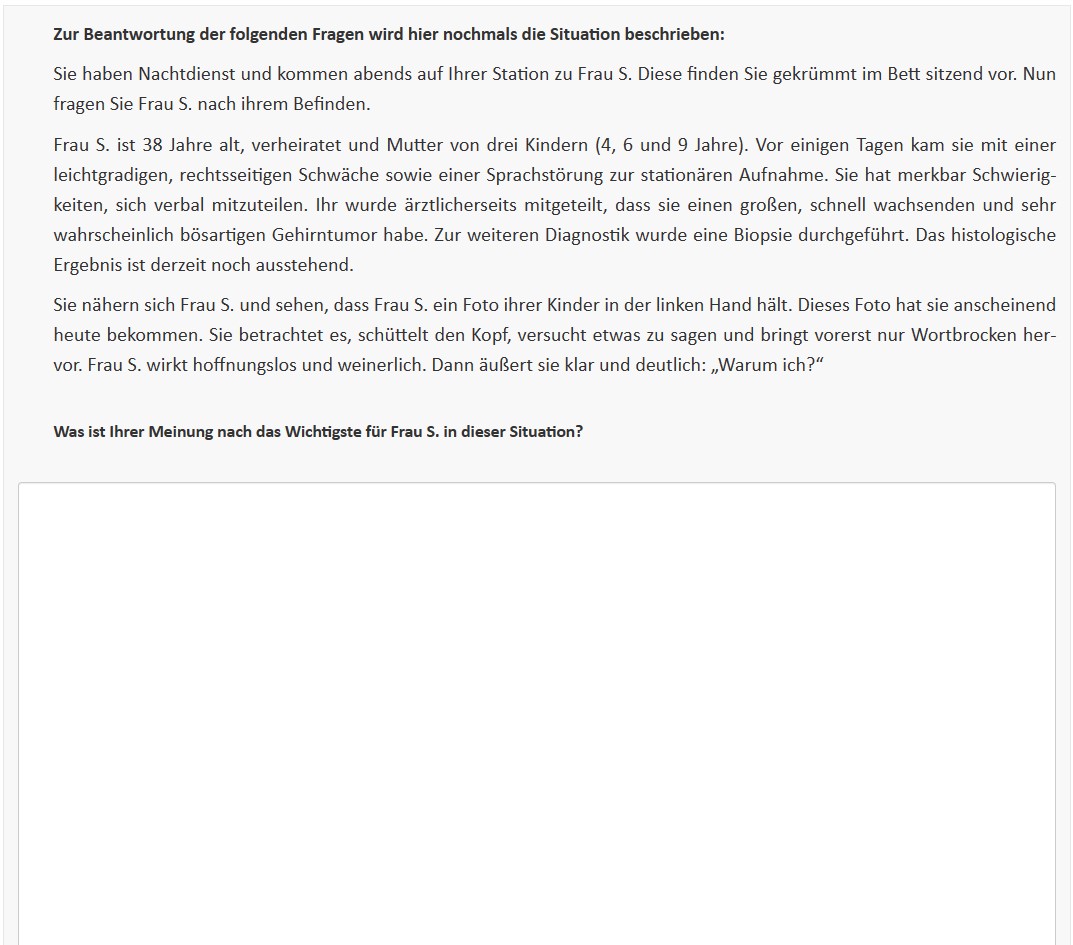

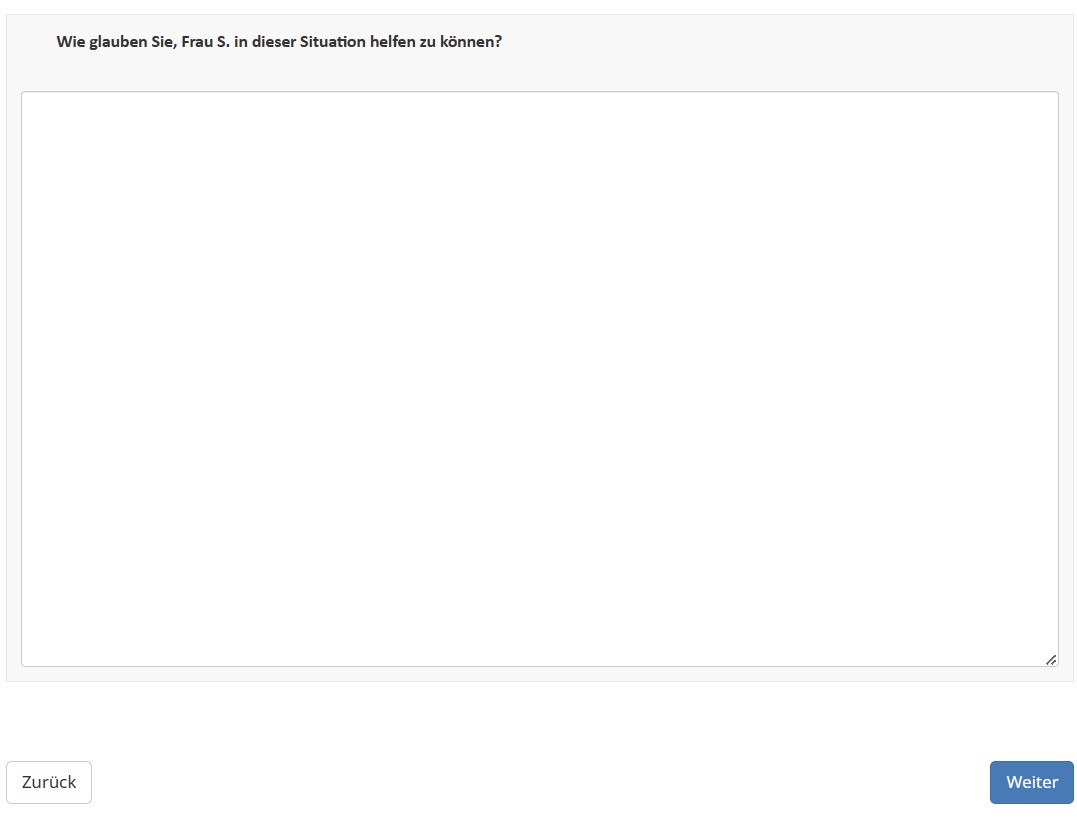
**

**
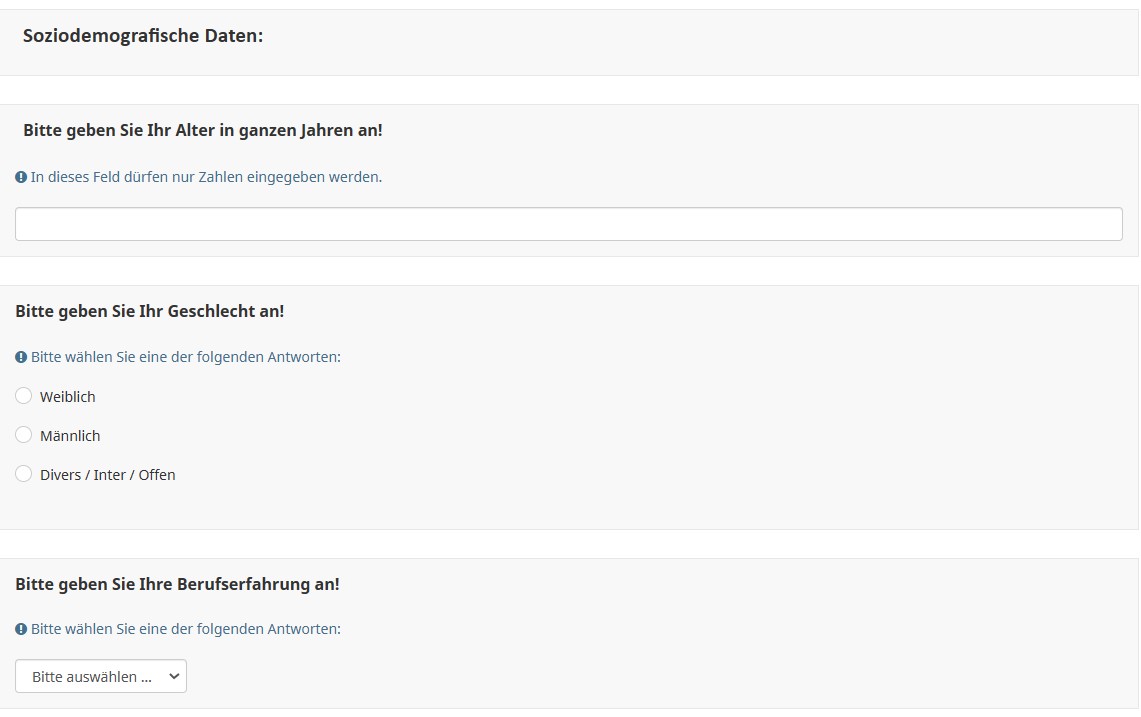

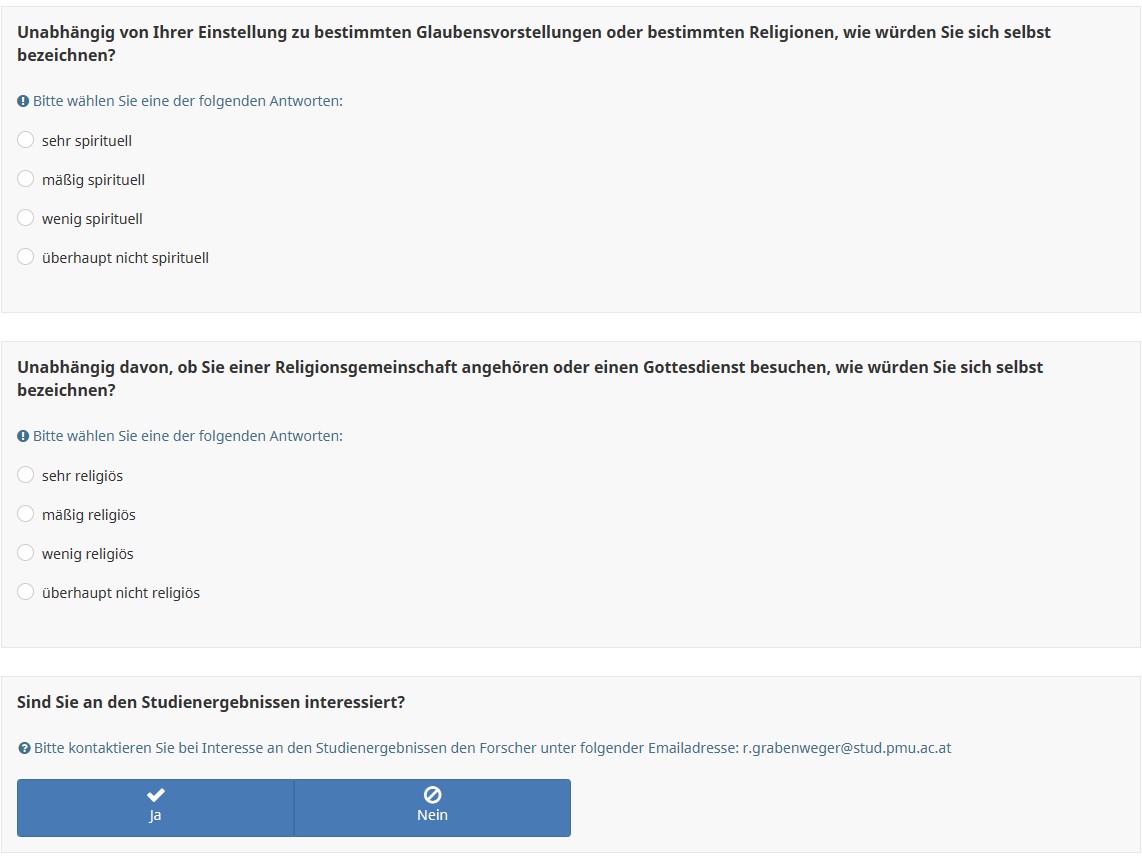

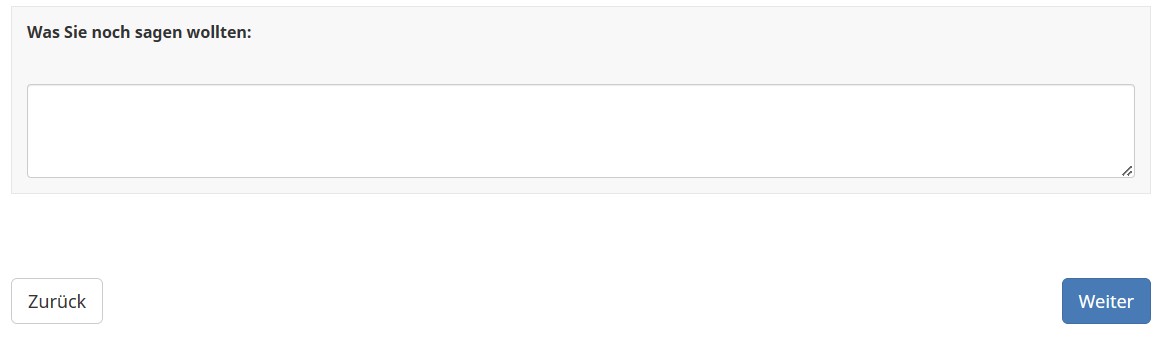
**

**
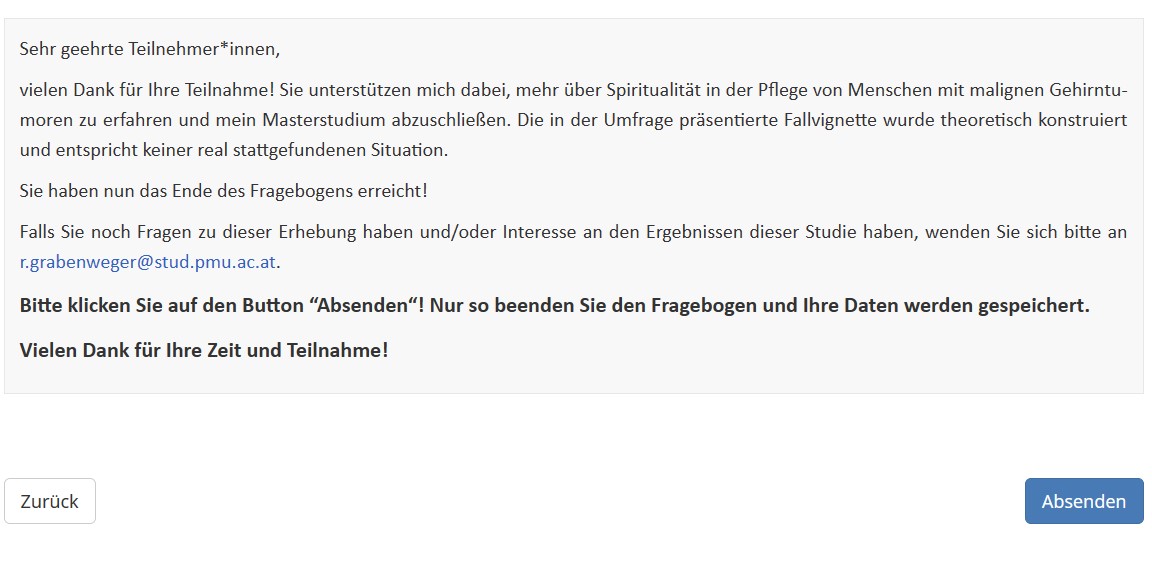
**


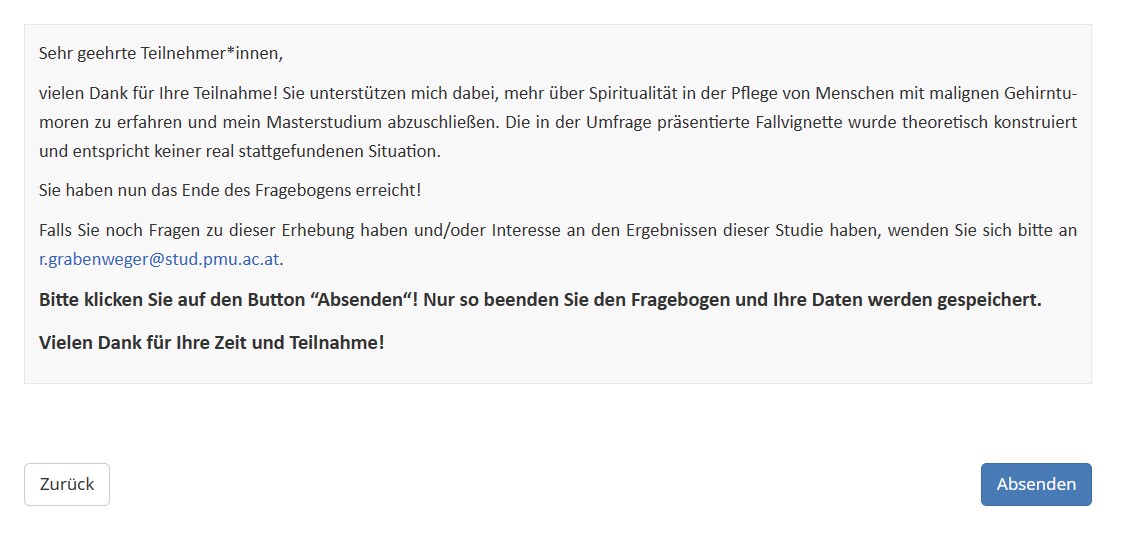

Supplement: Supplementary file 2 — Supplementary file2 (DOCX 988 KB) [file 10943_2025_2278_MOESM2_ESM.docx]
