# Supplementary material for: ‘Nurses as Gatekeepers’: Nurses’ Responses to Spiritual Needs of Patients with Primary Malignant Brain Tumors in Austria—Analysis of a Qualitative Vignette Study
Source: J Relig Health. 2025 Feb 21;64(2):732–53. doi: 10.1007/s10943-025-02278-7 (PMC11950041; doi:10.1007/s10943-025-02278-7)
Supplement: Supplementary file 3 — Supplementary file3 (DOCX 20 KB) [file 10943_2025_2278_MOESM3_ESM.docx]

Appendix 3: Original Data Extracts and their Translations

| **Original** | ***Translation*** |
| --- | --- |
| Ja, es kommen immer wieder zu Situationen in denen sich Patienten, Angehörige und Pflegende fragen "Warum trifft es mich? /Trifft es sie/ihn?". Im [s]tationären Alltag war nicht explizit eine junge Mutter dreier Kindern dabei. Aber ein junger Vater oder Pat. die kurz nach Pensionsantritt die Diagnose bekamen und jetzt eigentlich nochmal das Leben "genießen" wollten. (65, Pos. 1) | *Yes, there are always situations in which patients, relatives and caregivers ask themselves "Why does it happen to me? /Does it happen to her/him?". In inpatients’ everyday life, there was not explicitly a young mother with three children. But a young father or patients who were diagnosed shortly after retirement and actually wanted to "enjoy" life once again. (65, pos. 1)* |
| Ich habe mit der Pat. viele entlastende Gespräche geführt und sie dabei unterstützt die Informationen von [ä]rztlicher Seite zu verstehen und jederzeit bei Fragen zu Verfügung zu stehen […].  Das Gefühl nicht alleine zu sein und in dem Strudel aus Gefühlen und Informationen nicht unterzugehen und auch Zeit lassen die Gefühle und Eindrücke zu verarbeiten. (5, Pos. 2-3) | *I had many defusing conversations with the patient and helped her to understand the information from the doctor and was constantly available to answer questions [...].*  *The feeling of not being alone and not getting lost in the whirl of feelings and information and also allowing time to process the feelings and impressions. (5, pos. 2-3)* |
| Ja war ich schon mehrmals, als ich mit meinem Beruf angefangen habe 2007 war ich in diesen Situationen immer selbst sehr sensibel und musste aufpassen um nicht mitzuweinen, mittlerweile habe ich aber gelernt mich abzukapseln und Arbeit als Arbeit zu sehen. Dennoch fällt es mir immer noch schwer, besonders wenn die Patienten gleich alt wie ich oder jünger sind und vorher ein glückliches und zufriedenes Leben hatten. (62, Pos. 1) | *Yes, I have been several times, when I started my job in 2007 I was always very sensitive in these situations myself and had to be careful not to cry with the patients, but in the meantime I have learned to isolate myself and see work as work. Nevertheless, I still find it difficult, especially when the patients are the same age as me or younger and previously had a happy and satisfying life. (62, pos. 1)* |
| Nachdem die Pat einiges verweigerte und ich trotzdem noch aus zeitlichen Gründen ins nächste Zimmer musste, versicherte ich ihr, dass sie sich trotzdem jederzeit melden kann, wenn sie etwas brauchen würde. (24, Pos. 1-2) | *After the patient refused my suggested help and I still had to go to the next room for time reasons, I assured her that she could contact me at any time if she needed anything. (24, pos. 1-2)* |
| solche Ereignisse kommen in meinem beruflichen Alltag relativ oft vor.  Allerdings kommt es meiner Erfahrung nach auch sehr stark darauf an, welchen Raum man als Mensch/Pflegeperson solchen Situationen zugesteht.  Also wieviel Gespür, jenseits von sichtbaren Zuständen, und Empathie ich einbringe und auch Gesprächsbereitschaft nonverbal signalisiere. (47, Pos. 1-3) | *Such events occur relatively often in my everyday working life.*  *However, in my experience, it also depends very much on how much space you as a person/nurse allow for such situations.*  *In other words, how much sensitivity, beyond visible conditions, and empathy I bring with me and also signal non-verbally a willingness to talk. (47, pos. 1-3)* |
| Gesprächsbereitschaft signalisieren, Zeit nehmen, Zeit geben, Erzählen lassen (27, Pos. 3) | *Signaling willingness to talk, take time, give time, let people talk (27, pos. 3)* |
| Ich versuche dem Patienten auf Augenhöhe zu begegnen und mir ihre Sorgen aufmerksam anzuhören, biete wenn nötig psychologische Betreuung an und organisiere diese zeitnah. Meist ist es für den Betroffenen schon sehr wertvoll eine Gesprächspartner zu haben der sie ernst nimmt.  Ehrlichkeit im Gespräch ist sehr wichtig  Ein Gesprächspartner der sich Zeit für ihre Sorgen nimmt (10, Pos. 1-3) | *I try to meet the patient at eye level and listen carefully to their concerns, offer psychological support if necessary and organize it as soon as possible. It is usually very valuable for the affected person to have someone to talk to who takes them seriously.*  *Honesty is very important in this conversation.*  *A contact person who takes the time to listen to their concerns (10, pos. 1-3)* |
| - wenn sie es zulässt - sich zu ihr setzten, mit ihr reden - oder auch mal nichts sagen (26, Pos. 7) | *- if she allows it - sit down with her, talk to her - or sometimes say nothing (26, pos. 7)* |
| Ich finde es wichtig, dass sich die PP nicht selbst mit den Gesprächen überfordert fühlt und sich auch zurückziehen kann.  Andere Berufsgruppen wie Psychologen und Psychiater werden auf Wunsch des Patienten immer miteinbezogen und entlasten auch das Pflegepersonal. (55, Pos. 11-13) | *I think it's important that the nurse doesn't feel overwhelmed by the conversations and can also withdraw.*  *Other professional groups such as psychologists and psychiatrists are always involved at the patient's request and reduce the burden for the nursing staff. (55, pos. 11-13)* |
| an Ärzte weitergeleitet, um eine psychol. Unterstützung zu bekommen (39, Pos. 1) | *referred to doctors to get psychol. support (39, Pos. 1)* |
| + hinzuziehen von [p]sychologischen Diensten bzw. speziell ausgebildeten Personen inkl. Sozialarbeit (2, Pos. 5) | *+ Consult [p]sychological services or specially trained persons incl. social work (2, item 5)* |
| Nach dem Gespräch würde ich den Arzt kontaktieren, um ihr evt. etwas Beruhigendes zu geben. (64, Pos. 3) | *After the interview, I would contact the doctor to give her something reassuring if necessary. (64, pos. 3)* |
| Medikamente lehnte sie ab, Psychologisches und Psychiatrisches Konsil fand am nächsten Tag statt. (46, Pos. 3) | *She refused medication, psychological and psychiatric consultation took place the next day. (46, pos. 3)* |
| Später kann man den Psychologen oder/und den Psychiater hinzuziehen. (55, Pos. 10) | *Later, the psychologist and/or psychiatrist can be consulted. (55, Pos. 10)* |
| biete wenn nötig psychologische Betreuung an und organisiere diese zeitnah (10, Pos. 1) | *offer psychological support if necessary and organize this promptly (10, item 1)* |
| Kliniksozialarbeit und Psychologie wird angeboten und ggf. hinzugezogen (36, Pos. 2) | *Clinic social work and psychology are offered and called in if necessary (36, Pos. 2)* |
| Den Patienten nahelegen professionelle Gespräche in Anspruch zu nehmen (CL- Dienst) (61, Pos. 1) | *Encourage patients to make use of professional counseling (CL service) (61, Pos. 1)* |
| Wenn die Patientin gläubig ist die Seelsorge zu kontaktieren. (61, Pos. 3) | *If the patient is religious to contact the pastoral care. (61, pos. 3)* |
| Angebot einer Psychologie, und/oder eines Seelsorgers (insofern gläubige Personen), (27, Pos. 1) | *Offer of psychology and/or pastoral care (if the patient is religious), (27, pos. 1)* |
| Unsere Aufgabe ist es[,] zuzuhören und bei Überforderung den an der Station klinisch tätigen Psychologen miteinzubeziehen. (55, Pos. 6) | *'Our job is[,] to listen and to involve the psychologist working clinically on the ward if we are overwhelmed.' (55, item 6)* |
| Ich finde es wichtig, dass sich die [Pflegeperson] nicht selbst mit den Gesprächen überfordert fühlt und sich auch zurückziehen kann.  Andere Berufsgruppen wie Psychologen und Psychiater werden auf Wunsch des Patienten immer miteinbezogen und entlasten auch das Pflegepersonal. (55, Pos. 11-13) | *I think it's important that the [nurse] doesn't feel overwhelmed by the conversations and can also withdraw.*  *Other professional groups such as psychologists and psychiatrists are always involved at the patient's request and also relieve the nursing staff. (55, pos. 11-13)* |
